# The study design and rationale of the randomized controlled trial: translating COPD guidelines into primary care practice

**DOI:** 10.1186/1471-2296-14-56

**Published:** 2013-05-06

**Authors:** Donna R Parker, Charles B Eaton, David K Ahern, Mary B Roberts, Caitlin Rafferty, Roberta E Goldman, F Dennis McCool, Joseph Wroblewski

**Affiliations:** 1 Family Medicine and Epidemiology, Alpert Medical School of Brown University, Providence, RI, 02912, USA; 2 Center for Primary Care and Prevention, Memorial Hospital of Rhode Island, 111 Brewster Street, Pawtucket, RI, 02860, USA; 3 Abacus Health Solutions, 1210 Pontiac Avenue, Cranston, RI, 02920, USA; 4 Program in Behavioral Informatics and eHealth, Brigham & Women's Hospital/Harvard Medical School, Boston, MA, 02115, USA; 5 Department of Family Medicine, Alpert Medical School of Brown University, Providence, RI, 02912, USA; 6 Department of Medicine, Alpert Medical School of Brown University, Providence, RI, 02912, USA; 7 Memorial Hospital of Rhode Island, 111 Brewster Street, Pawtucket, RI, 02860, USA

**Keywords:** COPD, Guidelines, Randomized Clinical Trial, Primary care

## Abstract

**Background:**

Chronic obstructive pulmonary disease (COPD) is a progressive, debilitating disease associated with significant clinical burden and is estimated to affect 15 million individuals in the US. Although a large number of individuals are diagnosed with COPD, many individuals still remain undiagnosed due to the slow progression of the disorder and lack of recognition of early symptoms. Not only is there under-diagnosis but there is also evidence of sub-optimal evidence-based treatment of those who have COPD. Despite the development of international COPD guidelines, many primary care physicians who care for the majority of patients with COPD are not translating this evidence into effective clinical practice.

**Method/Design:**

This paper describes the design and rationale for a randomized, cluster design trial (RCT) aimed at translating the COPD evidence-based guidelines into clinical care in primary care practices. During Phase 1, a needs assessment evaluated barriers and facilitators to implementation of COPD guidelines into clinical practice through focus groups of primary care patients and providers. Using formative evaluation and feedback from focus groups, three tools were developed. These include a computerized patient activation tool (an interactive iPad with wireless data transfer to the spirometer); a web-based COPD guideline tool to be used by primary care providers as a decision support tool; and a COPD patient education toolkit to be used by the practice team. During phase II, an RCT will be performed with one year of intervention within 30 primary care practices. The effectiveness of the materials developed in Phase I are being tested in Phase II regarding physician performance of COPD guideline implementation and the improvement in the clinically relevant outcomes (appropriate diagnosis and management of COPD) compared to usual care. We will also examine the use of a patient activation tool - *‘MyLungAge*’ - to prompt patients at risk for or who have COPD to request spirometry confirmation and to request support for smoking cessation if a smoker.

**Discussion:**

Using a multi-modal intervention of patient activation and a technology-supported health care provider team, we are testing the effectiveness of this intervention in activating patients and improving physician performance around COPD guideline implementation.

**Trial registration:**

ClinicalTrials.gov, NCT01237561

## Background

Chronic Obstructive Pulmonary Disease (COPD) is a serious public health problem and is estimated to affect 15 million individuals in the United States [1]. Although the prevalence of COPD is increasing, the true burden of the condition is underestimated because COPD runs an insidious course and often results in an undiagnosed initial phase [2]. Consequently COPD is usually not recognized until it is “clinically apparent and moderately advanced” [3] and, in fact, COPD is most often diagnosed in the fifth or sixth decade even though the disease may actually start much earlier in life [4,5].

A joint activity between the National Heart, Lung and Blood Institute and the World Health Organization helped to develop and publish the Global Strategy for the Diagnosis, Management, and Prevention of Chronic Obstructive Lung Disease (GOLD) aimed at increasing awareness of COPD among health care providers and the general public and to provide comprehensive treatment guidelines with the goal of decreasing COPD related morbidity and mortality [6,7]. Despite the development of GOLD guidelines which provide guidance for appropriate prevention and management strategies, substantial gaps exist between the development of the guidelines and their implementation and dissemination in clinical practice. Consequently, COPD, which is a systemic disease with symptoms that overlap with other respiratory illnesses, remains underdiagnosed and undertreated [8-10].

The GOLD COPD guidelines have been developed to emphasize the importance of performing spirometry to make a firm diagnosis of COPD as well as to help stage COPD severity and guide specific treatment steps [11]. The guidelines recommend performing spirometry on all individuals with a history of repeated exposure to environmental pollutants and/or cigarette smoke exposure, a family history of COPD, or the presence of a chronic cough, sputum production, or shortness of breath. It has been suggested that “spirometry can be incorporated into family medicine practice with acceptable levels of technical adequacy and accurate interpretations” [12] based on the fact that improvements in spirometry equipment provide immediate feedback related to technical adequacy. We were interested in examining whether spirometry testing could be incorporated into primary care practice and potentially impact both the diagnosis and subsequent management of COPD. For the proposed study, we have provided office spirometers with adequate technical training to both intervention and usual care practices.

Current data suggest that there is underutilization of spirometry for detection and diagnosis of COPD [13]. Various reasons given by primary care physicians for not utilizing spirometry include: limited access to spirometry, uncertainty about the impact of the test, inadequate or lack of reproducibility of patient effort, lack of provider training, difficulty in interpreting results, reimbursement issues, and time constraints in busy practice settings [13,14]. Despite dissemination of the newer recommendations in an effort to increase primary care physician’s utilization of COPD guidelines, many primary care physicians remain unaware of COPD guidelines and the diagnosis of COPD continues to be based on clinical findings alone [15]. In summary, it appears that many patients with COPD are not diagnosed or staged with spirometry and therefore undertreated. Given this treatment gap, primary care providers will be the focus of the enhanced COPD guideline implementation and will allow us to examine whether a multi-modal intervention tailored to primary care practices will improve the care of patients with COPD.

## Methods/Design

**Figure 1:**
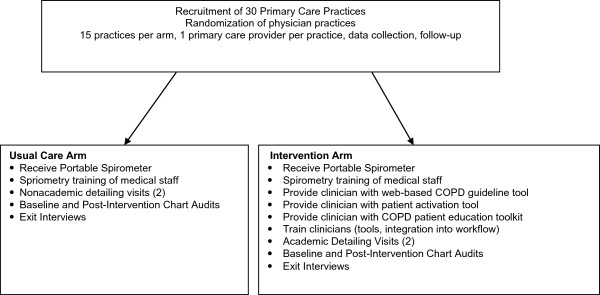
Overview of the RCT study design in phase II of translating the COPD guidelines into primary care practice.

This 5 year, National Institutes of Health (NIH) funded study is evaluating the translation of the Gold COPD guidelines into primary care practice. This study is based upon the premise that an informed, activated patient will interact with a prepared, proactive team to improve COPD diagnosis and management [16-20]. During Phase I, a needs assessment evaluated barriers and facilitators to implementation of COPD guidelines into clinical practice through focus groups of primary care patients and providers. Using formative evaluation and feedback from the focus groups, three tools were developed, refined and pilot tested. These include: 1) a patient activation tool on an iPad platform, 2) a Lung Age decision support tool to be used by primary care providers, and 3) a COPD patient education toolkit. During Phase I, procedure manuals, study protocols, and data collection instruments were also developed and pilot tested. During Phase II, a block, randomized design cluster trial (RCT) is currently underway with 30 primary care practices throughout the state of Rhode Island and southeastern Massachusetts. The effectiveness of the materials developed in Phase I is being tested in Phase II regarding physician performance of COPD guideline implementation and the improvement in the clinically relevant outcomes (appropriate diagnosis and management of COPD) compared to usual care. We are also examining the use of the patient activation tool (iPad) - *‘MyLungAge*’ to prompt patients to talk with their health care provider regarding their lung health and risk for COPD (Figure 1).

For the RCT of primary care practices, the unit of randomization is the practice. Practice, provider, and patient level outcomes are being assessed that include: pre- and post-assessment of practice characteristics, provider characteristics, and patient information based on chart audits. Patient chart audits are based on two samples: 1) one is based on an identified sample of at-risk or COPD patients (to examine management of these patients) and 2) the other is based on a de-identified random audit of charts from all practices. The first audit based on an identified sample of at-risk or COPD patients, who gave Informed Consent, are being conducted in the pre- and post-intervention periods. A random chart audit of de-identified patient outcomes both pre- and post-intervention is being conducted to determine the degree of selection bias inherent in the informed consent process. In addition, semi-quantitative assessments of the reach of the intervention are being undertaken on a regular basis through counting the number of ‘hits’ on the *MyLungAge* (iPad) patient activation tool located in each of the intervention practices and the number of tests initiated on the spirometer for each of the intervention providers. To better understand the workflow issues, strengths and weaknesses of our implementation strategy, a qualitative study of physicians’ understanding and adherence to the COPD guidelines, and a qualitative study of patients’ attitudes and perceptions regarding acceptance of COPD management recommendations and COPD medication adherence are being undertaken one year post intervention. This study is approved by the Memorial Hospital of RI Institutional Review Board.

### Providers

We are performing a two-group RCT, testing the effectiveness of translating the COPD guidelines into clinical practice, with primary care physicians’ practices as the unit of randomization and evaluation. The intervention arm receives an intervention of academic detailing, an iPad-based patient activation tool that will be placed in the examination rooms, a netbook running a COPD guideline decision support tool, a COPD patient education toolkit including a website, and a portable spirometer and a printer. The usual care arm receives visits on the same schedule as the academic detailing visits, a website link to the GOLD Guidelines, a printer, and a portable spirometer. The intervention is of one-year duration. We are evaluating the use of the interactive tool and use of the patient activation tool through tracking software.

### Patients

A cover letter was sent to a random selection of eligible patients (patients 40 years or older who had been seen at least once in the past 2 years (active patients) by their primary care provider). The patients received information regarding the study, were informed that their provider was participating in the study, and that they would receive a telephone call by one of the research study staff to invite them to participate in the study, to ask them to grant permission for auditing of their medical record at baseline and one-year post intervention, and to obtain Informed Consent. During the telephone call, patients received additional information regarding the study, were asked to provide audio consent and completed a 15-minute telephone interview that included demographic and medical history data and information regarding the patient’s knowledge, skills and behaviors necessary to create an activated patient for managing their own health care (PAM-a patient activation measure) [21].

### Measures

Data collection for practices includes: type of practice (HMO, single specialty, etc.); degree of managed care; service options (dietitian, nurse counseling, etc.,); numbers of personnel (physicians, RN, LPN, Nurse Practitioners, medical assistants, physician assistants, clerical, other); use of non-physician personnel, use of ancillary services in office (office laboratory, phlebotomy, dietitian, consultants); ancillary services in the building; who returns phone calls (physician vs. other); record systems (computerized, written, dictated); physical environment (rural, urban, suburban), socioeconomic class of patients seen; office organization (evening hours, weekend hours); and volume of visits (number scheduled, number of unscheduled, total number of patients, number of patient care hours, number no shows). Data collection of providers includes: 1) demographic information (age, gender), years in practice, type of provider (physician, nurse practitioner, physician assistant), specialty (general practitioner, family physician, obstetrics/gynecology, medicine/pediatrics, general internist, other); 2) practice characteristics (number of office visits, nursing home visits, and home visits seen in a typical week); 3) self-report of preventive screening (cholesterol screening, glucose screening for diabetes, self-report of counseling for smoking cessation, diet, physical activity) and 4) self-reported familiarity with COPD guidelines and feelings about COPD guidelines. Patient information from medical records includes: 1) socio-demographic factors- age, gender, race/ethnicity, insurance status, pharmacy benefits, marital status, number of visits in past year; 2) COPD risk factors (tobacco smoke, occupational dusts and chemicals, smoke from home cooking and heating fuel); 3) co-morbid conditions (i.e., coronary heart disease, congestive heart failure, arrhythmias, hypertension, stroke, liver disease, renal disease, arthritis, diabetes, malignancies, Parkinson’s disease, depression, osteoporosis); 4) functional status; 5) cachexia; 6) medications; 7) vital signs-height, weight, waist circumference, body mass index; 8) laboratory- hemoglobin, hematocrit; 9) provider documentation of weight loss, dietary assessment, dietary treatment or referral, physical activity assessment, and treatment for smoking cessation advice/ counseling/referral; 10) indicators needed to perform spirometry: dyspnea (worse with exercise, persistent, increased effort to breathe), chronic cough, chronic sputum 11) provider documentation of the need for pharmacotherapy; 12) provider documentation of adverse effects of COPD medications; and 13) intervention specific outcomes- documentation of *MyLungAge* in the chart, using printed SOAP notes or patient education page notes, use of the study smoking cessation material, and documentation of referral to the *MyLungAge* website. Data collection by chart audit is performed at baseline and again one-year post intervention. In addition, charts are audited for family history of COPD, smoking status, chest x-rays, ECGs, α_1_-antitrypsin deficiency screening, COPD symptoms (prolonged or progressive cough, sputum production, persistent or progressive dyspnea), exposure to lung irritants, exposure to secondhand smoke, documentation of spiromety, documentation of pulmonary disease, COPD, cystic fibrosis, interstitial lung disease, sarcoidosis, tuberculosis, pulmonary hypertension, thoracic surgery, immunizations and vaccines. Adherence to the COPD guidelines is based on an adherence tool developed by a consensus panel of 6 individuals with expertise in COPD and will include information on documentation of: exposure to tobacco smoke and occupational dust and chemicals; smoking status; appropriate treatment, increase in symptoms, influenza and pneumococcal vaccine; need for oxygen therapy; and assessment of history of CVD or CVD risk factors.

### Sample size considerations

The primary aim of this RCT is to determine and compare the proportion of participants who are appropriately diagnosed with COPD between the intervention and usual care arm. The clusters (i.e. practices) are randomly assigned to the intervention or usual care arm. Taking the intra-cluster correlation into account in the planning phase of the study is critical since failure to do so may result in an underpowered study. The intra-cluster correlation (r) is estimated to be between 0.01-0.02 based on previous findings [22]. For this study, we enrolled 30 practices, 15 for each group. Using a two-side type I error of 5%, the projected number of random charts per practice needed for a power of 80% is 35 for r=0.01 and 52 for r=0.02. Similarly, we assume the estimated percentage of patients who were diagnosed with COPD varied from 6% for the control group and 12% for the intervention group. The selection of 100 charts per practice in the random retrospective chart audits gives us reasonable power for all projected COPD outcomes. A total of 3000 charts for the study provide greater power for multivariate analyses, detecting interaction effects, robustness to violations of model assumptions. Given that we have audited 3,604 baseline charts, we have increased efficiency to detect weaker relationships between variables.

### Statistical analyses

Initially, descriptive statistics such as frequency tables for each categorical variable, and minimum, maximum, range, median, mean and standard deviation for each continuous variable will be used to summarize the data as well as detect outliers, data entry mistakes, and missing values. Classical longitudinal plots will be used to identify trends over time in both practice and individual levels. Chi-squared tests, t-tests or non-parametric tests will be used to assess the effectiveness of the randomization procedures by comparing intervention and control groups on baseline variables. Data analyses will be performed using SAS 9.1.3 (SAS Institute, Inc, Cary, North Carolina).

Following intention-to-treat principles, all participants who have been randomized to the two conditions will be included in the analyses. We hypothesize that a greater proportion of patients in the intervention arm will be appropriately diagnosed with COPD after one year compared to the usual care arm. We will use both generalized linear models using GEE (generalized estimating equations) approach and generalized linear mixed models to evaluate the independent effect of intervention after adjusting for patient and provider level covariates, important demographic and confounding factors. To compare COPD guideline adherence to the treatment algorithm recommended for primary care physicians after a one-year intervention in the usual care arm compared to the intervention arm, we will examine the dichotomous variable of whether or not a provider will adhere to the COPD treatment algorithm after a one-year intervention.

### Outcomes

Outcomes are being measured by medical record audit of 125 randomly selected patients per practice at baseline and one year post intervention; a random chart audit of de-identified patient outcomes both pre- and post-intervention to determine the degree of selection bias inherent in the informed consent process; and a chart audit of those patients who gave informed consent on the iPad patient activation tool in the intervention practices. A total of 180 charts are being audited per practice.

#### Time plan

The development of the program began in July, 2009 and the study will be completed in June, 2014. Provider recruitment started in October, 2010 and was completed in March, 2012 and patient recruitment began in December, 2010 and was completed in June, 2012.

## Discussion

In order to effectively overcome COPD guideline implementation problems, we need to better understand why primary care providers are underdiagnosing and undertreating patients with COPD and the barriers and facilitators to more fully implement the COPD guidelines. This study has allowed us to incorporate the insights discovered through qualitative methods in Phase I into the development of the provider interactive decision support tool on the netbook combined with the academic detailing materials. In addition to better understanding provider barriers and facilitators to guideline implementation, understanding patient knowledge, attitudes and behavioral intentions regarding COPD diagnosis and management is crucial. This information has helped us in developing appropriate patient activation tools, patient education and self-management tools, and the patient/provider shared decision-making screen incorporated into the *MyLungAge* iPad tool. We are in the process of utilizing these tools developed in Phase I, in the cluster design clinical trial in Phase II to determine the effectiveness of these tools in improving COPD guideline implementation. The results of this RCT will provide us with valuable insight into how to enhance implementation of COPD guidelines in primary care practices.

## Abbreviations

COPD: Chronic Obstructive Pulmonary Disease; RCT: Randomized clinical trial; GOLD: Global Strategy for the Diagnosis, Management, and Prevention of Chronic Obstructive Lung Disease; ECG: Electrocardiogram; CVD: Cardiovascular disease; GEE: Generalized estimating equation.

## Competing interests

All authors declare that they have no competing interests.

## Authors’ contributions

DRP conceived the study, participated in the design and coordination, and drafted the manuscript. CBE helped in the conception and design of the study and helped draft the manuscript, DA participated in the study design and helped draft the manuscript, MBR performed the statistical analyses and helped draft the manuscript, CR helped in the coordination of all study activities and collection of data and helped draft the manuscript, RG, FDM, and JW helped in the design and helped draft the manuscript. All authors read and approved the final manuscript.

## Pre-publication history

The pre-publication history for this paper can be accessed here:

http://www.biomedcentral.com/1471-2296/14/56/prepub
